# Intestinal manometry: who needs it? 

**Published:** 2015

**Authors:** Gabrio Bassotti, Sara Bologna, Laura Ottaviani, Michele Russo, Maria Pina Dore

**Affiliations:** 1 *Gastroenterology & Hepatology Section, Department of Medicine, University of Perugia Medical School, Perugia, Italy*; 2 *Gastroenterology Section, Perugia General Hospital, Perugia, Italy*; 3 *Department of Clinical and Experimental Medicine, University of Sassari, Italy *; 4 *Baylor College of Medicine, Houston, Texas, USA*

**Keywords:** Intestinal, Manometry, Motility, Myopathy, Neuropathy

## Abstract

The use of manometry, i.e. the recording of pressures within hollow viscera, after being successfully applied to the study of esophageal and anorectal motor dysfunctions, has also been used to investigate physiological and pathological conditions of the small bowel. By means of this technique, it has been possible to understand better the normal motor functions of the small intestine, and their relationship and variations following physiologic events, such as food ingestion. Moreover, intestinal manometry has proved useful to document motor abnormalities of the small bowel, although recognition of altered patterns specific for a determinate pathologic condition is still unavailable. However, this technique often permits the detection of abnormal gut motility in patients with abdominal symptoms such as unexplained vomiting and diarrhea, and it is sometimes also useful to address therapeutic targeting.

## Introduction

 Manometric techniques are methods that detect pressure events within hollow viscera. After being successfully employed to study upper (esophageal) and lower (anorectal) motility, manometry has also been applied to investigate the small (1) and the large bowel motility (2). Concerning the small bowel, these techniques have been extremely important to better elucidate several physiological mechanisms and demonstrate the pathophysiological bases of motor dysfunction in some pathologic disorders. Until recently, manometric techniques were based on the use of multilumen recording probes (with the lateral orifices arranged in various conformations to record different portions of the bowel), in turn connected to pressure transducers and to infusion systems (3). These techniques are presently often being replaced by the use of solid-state catheters that do not need perfusion, and may be directly connected to a recording system (polygraph, computer) for automated analyses (4), even though the perfused systems still maintain their validity.

**Physiological aspects**

**Figure 1:**
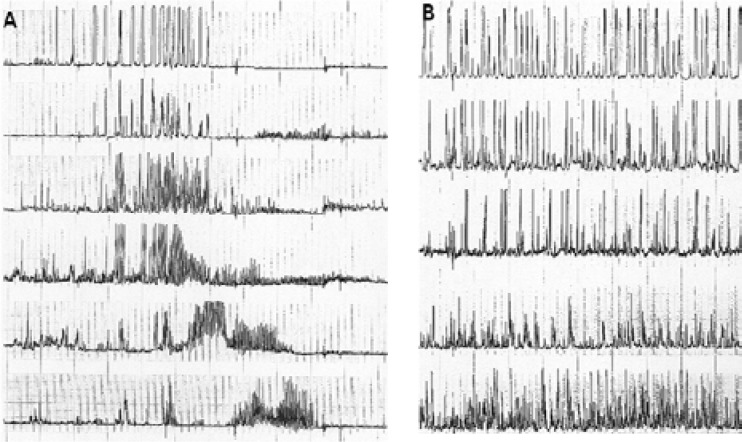
Antroduodenojejunal manometric recording in a healthy subject. A. During fasting, the three phases of the MMC are clearly identifiable; it is worth noting that the phase III is directed aborally from the antrum (first tracing) to the-jejunum (last tracing). B. After ingestion of a meal, a strong activation of contractile activity may be observed in all segments

In humans, the motor activity of the upper parts (stomach and small bowel) features specific patterns that mostly depend from individuals in the fasted or fed state (5). In fact, the fasting state is characterized by a pattern that displays cyclic timing and sweeps the bowel according to a oro-aboral programme (6). This pattern is named migrating motor complex (MMC) and is composed of three relatively well defined phases (7): phase I (in which with little or no contractile activity is present), phase II (in which intermittent and irregular contractions are documented), and phase III (the so-called activity front, which displays contractions occurring at a maximal rate, determined by the frequency of the slow waves in a specific segment) (Figure 1A). 

The three phases recur on average every 90 minutes, with phase III being relatively short (10% or less of the MMC), as well as phase I and phase II occupying each about 30%-80% of the cycle (8). It is worth remembering that the MMC may start physiologically from the distal esophagus (9), and therefore propagates to the terminal ileum (10). This inter-digestive cycle is interrupted by the ingestion of food, it is then replaced by randomly occurring frequent contractions (Figure 1B); this activity lasts about 2.5-8 hours and is progressively replaced by a new MMC (11). The duration of the fed motor activity strongly depends on the composition of the meal, lasts longer after a caloric than a non-caloric meal (12), and after fat-rich meals than after ingestion of other nutrients (13).

**Performing intestinal motility studies: when and in whom?**

The first important point to be stressed is that manometric studies of gastrointestinal motility should be preceded by an accurate exclusion of other organic and/or metabolic disorders by means of radiologic and endoscopic studies. The availability of less invasive radioisotopic techniques in some centers (14) may represent a reasonable alternative to manometric investigations, although these techniques are more expensive and have a little collateral biologic risk (15). 

Upper gastrointestinal manometry is chiefly used to investigate complaints of unexplained nausea, vomiting, abdominal pain and distention. in patients with an abnormal gastric emptying test in the absence of an etiological diagnosis (16, 17). However, it should be kept in mind that manometry in the stomach is reliable only to detect antral or antropyloric abnormalities, due to the large anatomical section of the viscus. When gastric emptying abnormalities are present, manometry is carried out to detect whether these abnormalities are limited to the stomach or belong to more generalized motility disorders (18). However, it must be stressed that intestinal manometry may reveal abnormal motor patterns in about only 50% of such patients (19-21). It is also worth noting that in most instances it is not possible to identify a specific motor pattern which can discriminate patients with severe motility-like dyspepsia from those with other diseases, or even from healthy individuals (22).

**Table 1:** Intestinal manometric abnormalities and their corresponding clinical situations

Manometric findings	Associated clinical situation
Sustained "minute" clustered contractions	Partial mechanical intestinal obstruction, Crohn’s disease
Repetitive, simultaneous, prolonged contractions of proximal small bowel	Subacute mechanical intestinal obstruction
Normally propagated but low amplitude contractions	Hollow visceral myopathies, intestinal pseudo-obstruction (myopathic forms), scleroderma
Abnormal propagation of antral and intestinal contractions	Intestinal pseudo-obstruction (neuropathic forms), severe dyspepsia, idiopathic gastroparesis, diabetes mellitus
Postprandial antral hypomotility	Diabetes mellitus, idiopathic or post-infectious gastroparesis, surgical vagotomy, dyspepsia
Minute clustered contractions (bursts)	Irritable bowel syndrome, intestinal pseudo-obstruction (neuropathic forms), food allergies, celiac disease, Whipple’s disease, Crohn’s disease, acute enteric infections, small bowel overgrowth

Manometric investigations may also help to identify the presence of abnormalities reconductable to neuropathic or myopathic disorders that affect the small bowel in both adult and pediatric patients (23-27). The therapeutic approach may vary according to the presence of neuropathic or myopathic features, with the myopathic ones being usually less responsive to a medical approach. These abnormalities might be important to define patients with chronic intestinal pseudo-obstruction (28,29).

Also, manometric techniques may reveal postsurgical motor abnormalities (30, 31). These abnormalities may help to characterize the patient’s symptoms in the postoperative period (32, 33). Further applications of intestinal manometry might help to define patients with severe intractable chronic constipation candidates for surgery, in whom the exclusion of motor abnormalities in the upper gut is important to avoid surgical failures or poor results (34).

More recently, intestinal manometry has been used to study patients with small bowel bacterial overgrowth. These studies have consistently shown the presence of small bowel motor abnormalities, suggesting that altered gut motility might likely predispose to the pathological growth of bacteria (35-37)

**Can intestinal manometry identify pathophysiological processes?**

**Figure 2:**
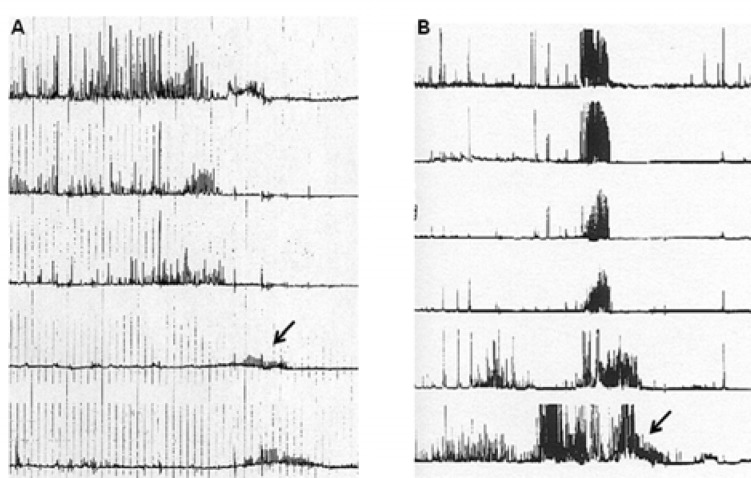
A. Manometric recording of a myopathic pattern. It is worth noting that the phase III of MMC features very low amplitude contractions (arrow). B. Manometric recording of a neuropathic pattern, featuring normal amplitude but uncoordinated (simultaneous) activity fronts and a sustained nonpropagated burst of activity in the last tracing (arrow

 Manometry is often able to provide evidence of a pathophysiological process; at the same time it is not usually diagnostic of a specific disease per se (38, 39). When available, manometry is useful to enforce the clinical suspicion of the presence of an abnormal motor activity by demonstration of a myopathic or neuropathic process. Manometry has a role in the process of diagnosis (Table 1). To date, it is possible to identify at least five main types of motor abnormalities in patients with suspected motility disorders using manometric techniques (40):

1) Patterns suggesting mechanical obstruction. These are represented by two events: a) a sustained (>30 minutes) postprandial pattern of "minute" clustered contractions separated by brief periods of motor quiescence (41); b) repetitive, simultaneous, prolonged contractions in the upper small bowel portions (42).

2) Generally low amplitude contractions, documented at several intestinal levels, and thought to be suggestive of a myopathic process. These low amplitudes (on average, below 15 mmHg) are mainly recorded in patients with hollow visceral myopathies or progressive systemic sclerosis (43, 44) (Figure 2A).

3) Normal amplitude, but “uncoordinated” (i.e., abnormally propagated) contractile activity in the gastric antrum and the small bowel, suggesting a neuropathic process. These motor abnormalities are usually present during phases II and III of the MMC (45) (Figure 2B); in addition, the persistence of a fasting pattern after eating a meal of >400 kcal is strongly suggestive of a neuropathic process (46). 

4) Postprandial antral hypomotility (infrequent contractions of normal amplitude). This pattern is frequently found in patients with diabetes mellitus, post-vagotomy, and postviral or idiopathic gastroparesis (47, 48).

5) Minute clustered contractions associated with abdominal pain firstly reported in patients with irritable bowel syndrome (49, 50). This kind of motor activity has been described in other subgroups of patients, such as those with untreated celiac disease (51) and food allergy (52), and it is common in healthy subjects (53).

**Limits of intestinal manometry**

Even though in selected subgroups of patients, neuropathic and myopathic motility patterns have been described (54), their pathological correlates are available only rarely. Therefore, it is often impossible to distinguish different pathological conditions only on the basis of manometric abnormalities (55). From a motor point of view, the human intestine seems to respond, in a monotonous manner following different pathophysiological noxae (see point 5 above). Thus, in the interpretation of manometric tracings caution should be a rule, since motility in the interdigestive state is extremely variable in human beings (56). Short (e.g., up to 2-3 hours) recording periods may show only one (or even none) motor event, such as that represented by the MMC (57). Therefore, in order to reduce the bias due to technical limitations, it is wise to carry out prolonged recordings (preferably for 24 hours by ambulant manometric techniques) (58). The recent introduction of automated analysis systems (59) will also help to better identify, define and establish the real values of intestinal manometric findings.

**Intestinal manometry: useful to establish therapeutic programs?**

Treating subgroups of patients with intestinal motor abnormalities may be complex, unsuccessful, and not infrequently frustrating (60). However, some evidences indicate that manometric techniques in selected individuals might help to find mechanisms likely responsible for the patient’s symptoms. Also, manometry may demonstrate the direct effect of possibly useful drugs on the motor abnormalities detected in these patients. For instance, it has been reported that octreotide injection stimulates MMC-like activity in scleroderma patients and reduces some of their symptoms (61), and other drugs have shown promising effects on intestinal motility (62, 63). Some authors tried to establish manometric findings as predictors of a therapeutic outcome. For instance, the persistence of fasting MMC may indicate a greater likelihood of response to prokinetic agents (64), and normalization of abnormal intestinal motility may predict the response to gluten-free diet, in both adults and children (65, 66). Again, the presence of intestinal motor abnormalities in patients with inactive Crohn’s disease could help management. The demonstration of the presence of a functional disorder may avoid g the risk of considering the ensuing symptoms as due to a disease’s relapse (67).

Finally, intestinal manometry may help to explain the gut motor responses to different food formulas (68, 69), thus providing useful information for the use of food manipulations during enteral nutrition.
